# *Bacillus velezensis* BVE7 as a promising agent for biocontrol of soybean root rot caused by *Fusarium oxysporum*

**DOI:** 10.3389/fmicb.2023.1275986

**Published:** 2023-10-20

**Authors:** Lei Sun, Wei Wang, Xue Zhang, Zhongchao Gao, Shanshan Cai, Shuang Wang, Yonggang Li

**Affiliations:** ^1^Heilongjiang Academy of Black Soil Conservation & Utilization, Harbin, China; ^2^College of Plant Protection, Northeast Agricultural University, Harbin, Heilongjiang, China

**Keywords:** soybean root rot, *Bacillus velezensis*, antifungal activities, biological control, growth-promoting effects

## Abstract

**Introduction:**

Soybean root rot (SRR), caused by *Fusarium oxysporum*, is a severe soil-borne disease in soybean production worldwide, which adversely impacts the yield and quality of soybean. The most effective method for managing crop soil-borne diseases and decreasing reliance on chemical fungicides, such as *Bacillus* spp., is via microbial biocontrol agents.

**Methods and Results:**

In this study, a soil-isolated strain BVE7 was identified as *B. velezensis*, exhibiting broad-spectrum activity against various pathogens causing soybean root rot. BVE7 sterile filtrate, at a concentration of 10%, demonstrated significant antifungal activity by inhibiting the conidial germination, production, and mycelial growth of *F. oxysporum* by 61.11%, 73.44%, and 85.42%, respectively, causing hyphal malformations. The antifungal compound produced by BVE7 demonstrated adaptability to a standard environment. The pot experiment showed that BVE7 suspension could effectively control soybean root rot, with the highest control efficiency of 75.13%. Furthermore, it considerably enhanced the activity of catalase, phenylalanine ammonia lyase, superoxide dismutase, and peroxidase in soybean roots, while also preventing an increase in malondialdehyde activity. By improving the host resistance towards pathogens, the damage caused by fungi and the severity of soybean root rot have been reduced.

**Discussion:**

This study presents the innovative utilization of *B. velezensis*, isolated from soybean roots in cold conditions, for effectively controlling soybean root rot caused by *F. oxysporum*. The findings highlight the remarkable regional and adaptive characteristics of this strain, making it an excellent candidate for combating soybean root rot in diverse environments. In conclusion, *B. velezensis* BVE7 demonstrated potential in effectively reducing SRR incidence and can be considered as a viable option for SRR management.

## 1. Introduction

Soybean is a significant food crop that provides a sustainable source of protein, oil, vitamins, and other essential nutrients for human consumption globally (Hu et al., 2016; Yang et al., 2023). As the primary soybean-producing region in China, Northeast China accounts for 50% of the country’s total soybean production (source: China National Data, https://data.stats.gov.cn/). It is crucial to reduce disease damage during soybean planting to enhance yields (Dorrance et al., 2003). In the early spring, when soybean seeding occurs, the damp environment is conducive to pathogen invasion, which can cause soybean root rot at different temperatures (Zitnick-Anderson and Nelson, 2015). *Fusarium* spp. including *F. redolens* in Minnesota (Bienapfl et al., 2010), *F. oxysporum*, *F. graminearum, F. solani*, *F. avenaceum*, *F. tricinctum*, *F. sporotrichioides*, *F. equiseti*, and *F. poae*in and *F. proliferatum* Ontario, Canada (Zhang et al., 2009, 2013; Chang et al., 2015), *F. commune*, *F. solani*, *F. tricinctum* and *F. fujikuroi* in the United States (Ellis et al., 2013; Detranaltes et al., 2021; Yan and Nelson, 2021), *F. oxysporum, F. brachygibbosum* and *F. fujikuroi* in China (Li et al., 2018; Zhao et al., 2020; Wang et al., 2021), cause soybean root rot (SRR), a soil-borne disease with a high risk of infection, significantly impacting soybean emergence, seedling growth, plant vigor, and yield losses (Nelson et al., 1999; Han et al., 2021). *F. oxysporum* is the dominant strain reported in the United States (Nelson et al., 1999; Cruz Jimenez et al., 2018), Northeast China (Li et al., 2018), and Ontario, Canada (Wang et al., 2004; Zhang et al., 2013).

SRR, caused by *F. oxysporum*, is a common soil-borne disease. Conventional methods for controlling root rot, such as seed dressing and leaf-spraying with chemical fungicides in the field, have been utilized to manage some Fusarium diseases (Zhang et al., 2000). However, given the impact of chemical fungicides on the ecological environment, human health, and pathogen resistance, there has been increased attention on the limitations of chemical control. Crop rotation with non-host plants is an effective measure for controlling SRR. Buhre and Kluth (2009) employed crop rotation of maize and beet to manage the incidence of beet root rot. Nonetheless, there are few land resources and accumulated temperatures in Northeast China, this measure may not be applicable in areas with continuous soybean cropping due to specific climatic conditions and economic considerations (Li et al., 2010). Therefore, there is an urgent need to identify a harmless and feasible strategy for suppressing the development of Fusarium root rot on soybean.

Soil is a living natural resource on which the sustainability of agricultural systems depends. Soil quality is attributed to multiple interactions between physical, chemical, and biological components, with microbial communities playing a crucial role in soil functionality (Janvier et al., 2007). In addition, the complexity of ecosystems is directly involved in the performance of soil system functions, determining its quality (Vezzani and Mielniczuk, 2009). Biological control of plant diseases using plant-associated bacteria or natural compounds of biological origin is now recognized as one of the most promising alternatives to the use of chemical fungicides (Etesami et al., 2023). Employing beneficial microorganisms for biological control is a noteworthy strategy that has significant potential for managing Fusarium root rot on soybean. Currently, chemical control remains the main approach for preventing and treating crop root rot. Yuan et al. (2011) effectively managed wheat root rot using difenoconazole. Tanni et al. (2016) successfully controlled the root rot of chickpea through the use of Bavistin 70 WP. Naqvi (2005) controlled root rot (*Phytophthora nicotianae*) in citrus soils with foselyl-Al and Metalaxyl. However, chemical control methods have negative impacts on ecological environment and human health, and are not conducive to the sustainability of agriculture. In comparison to the application of chemical agents, biological control measures can be both cost-effective and environmentally friendly, and can significantly inhibit the activity of soil-borne pathogens and induce plant resistance due to the natural and pathogenic origins of their biological control agents (BCAs) (Zhang et al., 2009; Amin et al., 2015; Raza et al., 2019). Furthermore, biological control provides long-lasting and sustainable effects in reducing the incidence and severity of root rot, ensuring the healthy growth of current and subsequent crops. Huang et al. (2013) found that the rhizosphere soil of healthy plants that survive in plots infected by plant pathogens is a good source for the isolation of BCAs. In recent years, numerous BCAs have been successfully isolated from rhizosphere soil, including *Bacillus* spp. (Xu et al., 2020), *Pseudomonas* spp. (Liu et al., 2019), *Trichoderma* spp. (Saravanakumar et al., 2017), and *Streptomyces* spp. (Faheem et al., 2015). Among these promising BCAs, *Bacillus* spp. produces stress-resistant spores and exhibits resistance to extreme conditions, fast reproduction, and strong colonization ability. It is amenable to artificial large-scale fermentation and cultivation, making it an ideal candidate for use as a biocontrol bacterium (Santoyo et al., 2012; Shafi et al., 2017; Fira et al., 2018). *Bacillus* spp., among the various biotic components, is a predominant bacterial genus with tremendous metabolic and genetic diversity, which enables it to play a crucial role in the soil ecosystem. Many *Bacillus* species have been found in different ecological niches. Globally developed and distributed commercial *Bacillus*-based preparations contain *B. amyloliquefaciens*, *B. cereus*, *B. licheniformis*, *B. megaterium*, *B. pumilus*, *B. subtilis*, *B. thuringiensis*, and *B. velezensis* species (Mazzola and Freilich, 2017; Etesami et al., 2023). *Bacillus* spp. populations can coexist with other bacterial populations in the soil and rhizosphere without any negative effects (Vardharajula et al., 2011; Radhakrishnan et al., 2017). Zhang et al. (2009) utilized *Bacillus subtilis* strains through seed and soil treatments to manage soybean root rot induced by *F. oxysporum* and *F. graminearum*. Therefore, *Bacillus* species were chosen as an alternative biocontrol factor for preventing and managing SRR caused by *F. oxysporumin* in this paper.

The research aimed to achieve the following objectives: (i) to screen and identify biocontrol bacteria for the management of SRR, (ii) to analyze the antifungal mechanisms of *B. velezensis*, a potential biological control bacterium, (iii) to investigate the impact of biocontrol bacteria on defense-related enzymes in soybean, and (iv) to evaluate the efficacy of employing biocontrol bacteria in the management of SRR.

## 2. Materials and methods

### 2.1. Isolation of bacterial strains

Three hundred and twenty-four bacterial strains were isolated from the rhizosphere soil of soybean fields located in Harbin (126.93° E, 45.77° N), China, using the soil dilution method (Li et al., 2020). Following a 72-h incubation period at 28°C, a single bacterial colony was selected and purified via repeated streaking on beef extract peptone medium (BPM) (Beijing Aoboxing Biology Technology Co., Ltd.) plates.

### 2.2. Screening of biological control bacteria against SRR

The targeted pathogen, *Fusarium oxysporum* isolate BLD3, was isolated and preserved by our team and eventually emerged as the primary population of SRR-causing pathogens in Northeast China, emerging as the predominant pathogen responsible for SRR in the area. Initial screening of antagonistic bacteria was accomplished using the confrontational approach (Birber et al., 1998). After incubating the bacterial strains on a nutrient agar medium (NA) plate for 48 h at a temperature of 28°C, a center of a potato dextrose agar (PDA) plate with a diameter of 9 cm was inoculated using the parallel streak method positioned at a distance of 3 cm. Following this, a disk of *F. oxysporum* with a diameter of 0.7 cm was inserted in the center of the PDA plate. Cultivating for 5 days at a temperature of 26°C with three replicates, measurements were taken for the maximum and minimum radii of *F. oxysporum* colonies to determine the antagonistic activity of the tested bacterial strains (Wang et al., 2020). Strains that displayed the most significant ratio of the longest-to-shortest radius were selected for further in-depth study.

### 2.3. Identification of biocontrol bacterium BVE7

The morphological features of BVE7 were observed on nutrient agar (NA), while the physiological and biochemical characteristics were measured based on standard protocols by Schaad et al. (2001), Buchanan (1984), and Dong and Cai (2001). Based on the aforementioned references, we had chosen these physiological and biochemical parameters to assist in the identification of the genus and species of biocontrol bacteria, including tests for gram stain, lactose utilization, catalase activity, sucrose utilization, aerobism, mannitol fermentation, glucose fermentation, V.P. test, fructose fermentation, gelatin liquefaction, arabinose utilization, mannose fermentation, xylose utilization, casein hydrolysis, sorbitol utilization, phaseomannite utilization, starch hydrolysis, malonate utilization, cellobiose fermentation, rhamnose utilization, M.R. test, maltose fermentation, galactose fermentation, and nitrate reduction using commercially available physiological and biochemical test kits (Guangdong Huankai Microbial Sci.&Tech. Co., Ltd., Guangdong, China).

To identify BVE7, genomic DNA was extracted using a Tiangen Genome Extraction Kit (Tiangen Biotech, Beijing, China). Bacterial universal primers, 27F (5′-AGAGTTGATCCTGGCTCAG-3′) and 1492R (5′-GGTTACCTTGTTACGACTT-3′), were employed to amplify partial sequences of the 16S rRNA gene. The genomic template was amplified by PCR in a 50-μl reaction volume consisting of 25-μl PCR Master Mix (2X) (Invitrogen, Carlsbad, CA, United States), 2.0 μL of 10 mM forward primer, 2.0 μL of 10 mM reverse primer, 2 μL of template DNA, and 19 μL of nuclease-free water. The PCR program consisted of an initial denaturation at 94°C for 5 min, followed by 36 cycles of denaturation at 94°C for 1 min, annealing at 58°C for 1 min, and extension at 72°C for 1.5 min; and a final extension at 72°C for 10 min. The amplified product was sequenced by Shanghai Biological Engineering Co., Ltd. (Shanghai, China). Phylogenetic trees of BVE7 were constructed using Mega 6.0 software (Mega Limited, Auckland, New Zealand) based on the neighbour-joining (NJ) method (Nei and Kumar, 2000).

### 2.4. Antifungal spectrum

Using the described confrontation technique, the antifungal efficacy of BVE7 against 10 strains of SRR-causing pathogenic fungi, including *F. tricinctum*, *Diaporthe longicolla*, *F. acuminatum*, *Bipolaris zeicola*, *Chaetomium globosum*, *F. verticillioides*, *Botrytis cinerea*, *F. solani*, *Clonostachys rosea*, *F. chlamydosporum*, was assessed. These pathogenic fungi were obtained from the plant pathology laboratory of Northeast Agricultural University in China. The measurement and evaluation techniques were identical to those outlined in Section 2.2.

### 2.5. Determination of antagonistic mechanism of BVE7

BVE7 was activated at 28°C for 24 h with agitation at 180 rpm and subsequently inoculated into Luria-Bertani (LB) liquid medium with a liquid loading of 200 mL·L − 1 in a 500-mL Erlenmeyer flask under identical conditions for six days. The aseptic filtrate of BVE7 was obtained through an aseptically sterilized bacterial filter with a pore size of 0.22 μm (YY3014236, Millipore, United States) (Li et al., 2020).

An aseptically filtered solution of BVE7 was added to Potato Dextrose Agar (PDA) medium at final concentrations of 1, 5, and 10%, with three replicates each. An equivalent volume of liquid Luria-Bertani (LB) medium was added as a control. This experiment was conducted twice. A 7 mm diameter disc of *F. oxysporum* mycelium grown on PDA medium for 120 h was transferred to the center of the PDA plate and incubated for another 120 h at 26°C. The diameter of *F. oxysporum* colonies was measured to evaluate the inhibitory effect of BVE7 on its growth.

*Fusarium oxysporum* was cultivated on PDA plates at 26°C until the colony reached a diameter of 4 cm. The medium without hyphae was discarded, and then 20 mL of aseptic BVE7 filtrate was added to the PDA plates with the colony at concentrations of 1, 5, and 10%. 20 mL of LB liquid medium was used as a control with three replicates. After a 20-min incubation period, the mycelium was carefully removed. Following 72 h of incubation at 26°C, 20 mL of sterile water was poured into the plates to wash the conidia, and conidia concentration was recorded using a haemocytometer (Li et al., 2020). The experiments were repeated twice to ensure accuracy.

*Fusarium oxysporum* was cultivated on PDA media at 26°C for 5 days followed by washing with sterile distilled water (SDW) to obtain conidia. A haemocytometer was used to adjust the conidial suspension to 1 × 10^8^ conidia/mL. BVE7 filtrate was then added to the conidial suspension at concentrations of 1, 5, and 10%, which was prepared using the same method as described earlier. For the control, conidial suspension amended with an equal volume of LB liquid medium was used. Each treatment was subjected to three replicates. The conidial suspensions were incubated at 25°C. Conidia germination (100 spores per treatment) was counted when the control’s conidial germination rate exceeded 60%.

*Fusarium oxysporum* was inoculated onto PDA plates and incubated at 26°C for 12 h. Fresh hyphae were then scraped and immersed in aseptic BVE7 filtrate at concentrations of 1, 5, and 10%. After 24 h, the morphology of the hyphae was observed under an optical microscope (Nikon 90i, Japan) in order to obtain precise results.

### 2.6. Stability of antifungal substances

Aseptic filtrates of BVE7, obtained through procedures outlined in section 2.5, were subjected to incubation in a water bath at temperatures of 20, 40, 60, 80, and 100°C, and 121°C. The effects of aseptic filtrates treated at varying temperatures were evaluated on the mycelial growth of F. oxysporum at a concentration of 5%.

The pH of the aseptic BVE7 filtrate was adjusted in increments of 1 unit from 3.0 to 12.0 using 0.1 M HCl or NaOH. The impacts of different pH treatments on BVE7 were determined by using the mycelial growth rate method.

The aseptic BVE7 filtrate was exposed to UV light at a distance of 1 cm and at a wavelength of 100 W/cm^2^ for 30, 60, and 90 min. The effects of the UV light exposure on BVE7 were evaluated utilizing the aforementioned method.

### 2.7. Evaluation of biological control efficacy of BVE7 under pot conditions

The specific procedures were carried out as follows: the biocontrol bacteria BVE7 were cultured in liquid LB medium for 48 h and subsequently diluted to a concentration of 1 × 10^8^ CFU/mL (where an OD of 0.1 at 600 nm is equivalent to 10^8^ CFU/mL) using 0.9% normal saline solution as a diluent for storage. Inoculum of *F. oxysporum* was prepared by inoculating sorghum seeds following the protocol described in our previous research (Li et al., 2018). The study included six treatments, each with three replicates. i was the control group with no treatment. Iiwas only inoculated with *F. oxysporum* iii involved irrigating 0.5 mL of bacterial suspension to the roots and *F. oxysporum* on the day of planting and 7 days later. iv used 1 mL of bacterial suspension per plant and *F. oxysporum*, while v used 1.5 mL per plantand *F. oxysporum*. vi involved spraying seeds with 43% tebuconazole at 0.14 mg/mL. The pots were placed in a greenhouse with a temperature of 23 ± 3°C. A total of fifty plants were inoculated with three repetitions, and after inoculation, conventional methods were utilized in managing the seeds (cv. Dongnong 52). The occurrence of SRR was then evaluated after 20 days.

The severity of the disease was evaluated by assessing the growth status of soybean roots using a scale ranging from 0 to 9 (Li et al., 2018): Where 0 = no symptoms; 1 = slightly darkening fibrous root, the aboveground portion grew well; 3 = slightly darkening taproot, the aboveground portion grew well; 5 = severe darkening taproot or hypocotyls erosion, the aboveground portion grew poorly; and 7 = root necrotized and infected plant dead. The experiment was conducted twice under identical conditions. Disease index (DI) for SRR was calculated, as follows:


$$\begin{array}{l} DI=\sum \left(\mathrm{number}\;\mathrm{of}\;\mathrm{diseased}\;\mathrm{plants}\;\mathrm{at}\;\mathrm{each}\;\mathrm{level}\times \mathrm{number}\;\mathrm{of}\;\mathrm{relative}\;\mathrm{ratings}\right)\\ \;/\left(\mathrm{total}\;\mathrm{number}\;\mathrm{of}\;\mathrm{surveyed}\;\mathrm{plants}\times \mathrm{highest}\;\mathrm{number}\;\mathrm{of}\;\mathrm{diseased}\;\mathrm{levels}\right)\times 100\text{.} \end{array}$$

### 2.8. Analysis of defense-related enzymes in soybean seedlings treated by the biocontrol bacterial strain BVE7

Soybean seedlings (10 plants per pot) grown for 15 days were inoculated with a suspension of BVE7 spores at a concentration of 1 × 10^8^ cfu/mL by root irrigation, with 1.5 mL per plant, while an equal amount of sterile water was used as a blank control. Each treatment was replicated three times. The treated plants were placed in a constant-temperature incubator maintained at 25°C, with a light/dark regime of 12/12 h and 85–95% relative humidity (RH). After 0, 12, 24, 48, 72 and 96 h of incubation, root tissues from the treated soybean were collected and analyzed for defense-related enzyme activity. The enzyme activities of catalase (CAT), malondialdehyde (MDA), phenylalanine ammonia-lyase (PAL), peroxidase (POD), and superoxide dismutase (SOD) were determined using appropriate kits (Suzhou Grace Biotechnology Co., Ltd., Suzhou, China) and calculated according to the fresh weight of the sample in units per kilogram (U kg-1).

### 2.9. Data analysis

All experiments were replicated twice under identical conditions. Statistical analysis was performed using SPSS Statistics 19.0 software (IBM Corporation, Armonk, NY, United States), and results were evaluated using ANOVA. Subsequently, statistically significant differences were observed between the means of the treatments using Duncan’s multiple range test (*p* < 0.05).

## 3. Results

### 3.1. Isolation and screening of biological control bacteria

**Figure 1:**
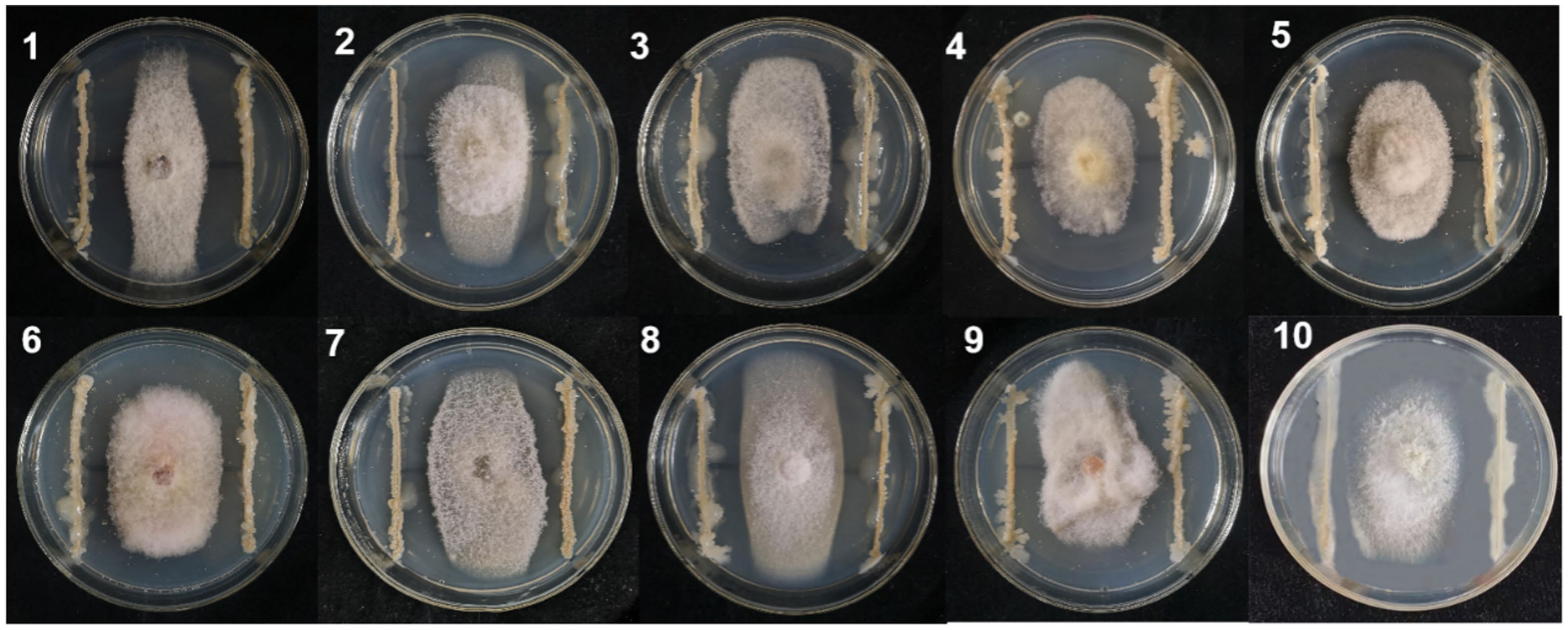
Antagonistic effect of biological bacteria BVE7 on the mycelial growth of pathogenic fungi on the fifth day after inoculation. 1. *Diaporthe longicolla*; 2. *Clonostachys rosea*; 3. *Bipolaris zeicola*; 4. *Chaetomium globosum*; 5. *Botrytis cinerea*; 6. *Fusarium acuminatum*; 7. *F. Solani*; 8. *F. tricinctum*; 9. *F. chlamydosporum*; 10. *F. verticillioides.*

A total of 324 strains extracted from the rhizosphere soil of soybean plants were screened for their antagonistic activity against *F. oxysporum*, the causal agent of SRR. Out of all the tested strains, a total of 24 demonstrated superior antagonistic activity in comparison to the remaining strains, with the BVE7 strain exhibiting the most prominent effect (Table 1) and was identified as a potential biocontrol agent with an average ratio of 2.50. Further evaluation of BVE7 in potted soybean plants demonstrated its excellent control efficacy against SRR. Accordingly, BVE7 was selected for subsequent experiments. Additionally, BVE7 displayed remarkable antagonistic activity against various pathogenic fungi causing SRR (Table 2 and Figure 1), including *Diaporthe longicolla*, *Clonostachys rosea*, *Bipolaris zeicola, Chaetomium globosum*, *F. solani*, *F. tricinctum*, and *F. verticillioide*s.

**Table 1:** Twenty-four bacterial strains screened for their strong antagonistic effects against *Fusarium oxysporum* causing root rot in soybeans.

Antagonistic Strains No.	Maximum/Minimum radius^a^	Antagonistic Strains No.	Maximum/Minimum radius^a^
BVE7	2.50	B-13	2.01
F3	2.47	12–2	2.01
107	2.47	24	2.00
H-18	2.41	A27	1.99
B6	2.35	26	1.97
21	2.35	22	1.97
18	2.35	23	1.94
F5	2.13	25	1.76
19	2.09	94	1.76
30	2.07	S2	1.75
91	2.06	12	1.67
13	2.04	11	1.54

a Values in the column indicate mean of the maximum/ minimum radius of the pathogens.

**Table 2:** Determination of antagonistic fungal spectrum suppressed by biological bacteria BVE7 *in vitro*.

Strains No.	Mean ± SE^a^	Strains No.	Mean ± SE^a^
*Diaporthe longicolla*	2.67 ± 0.08a	*Fusarium acuminatum*	1.47 ± 0.09 g
*Clonostachys rosea*	2.18 ± 0.11bcd	*F. solani*	2.10 ± 0.04cde
*Bipolaris zeicola*	2.00 ± 0.10de	*F. tricinctum*	2.31 ± 0.05b
*Chaetomium globosum*	2.01 ± 0.13de	*F. chlamydosporum*	1.93 ± 0.12ef
*Botrytis cinerea*	1.82 ± 0.09f	*F. verticillioides*	2.31 ± 0.05b

a Values in the column indicate mean ± standard error (SE) of the maximum/ minimum radius of the pathogens on the fifth day after inoculation.

Values followed by different letters are significantly different according to Duncan’s multiple range tests (*p* < 0.05).

### 3.2. Identification of isolate BVE7

**Figure 2:**
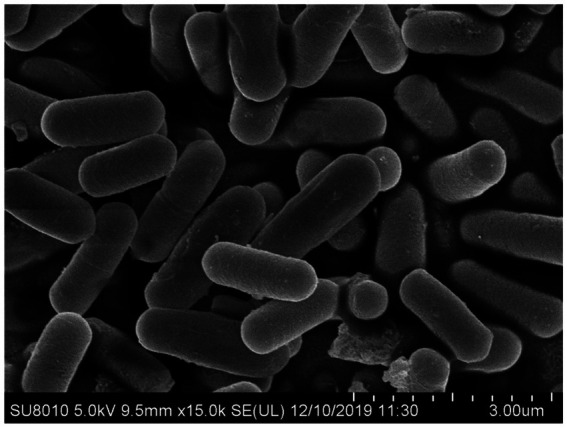
Scanning electron micrograph depicting the cellular morphology of *Bacillus velezensis* BVE7 cultivated for 24 h at 30°C on nutrient agar medium.

**Figure 3:**
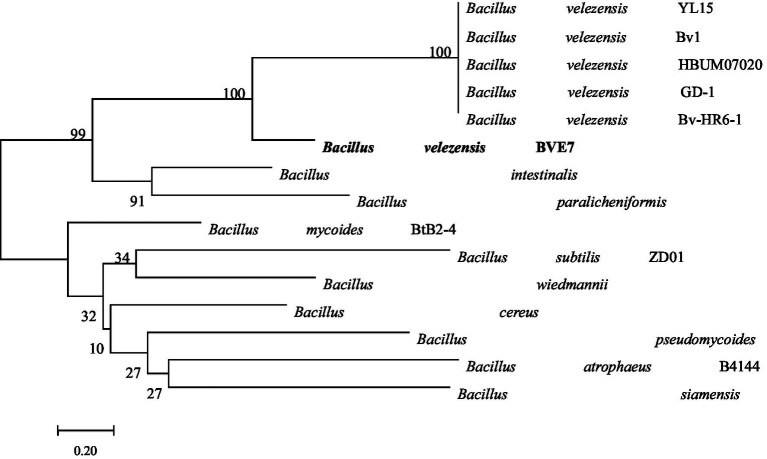
Phylogenetic tree depicting the identification of *Bacillus velezensis* isolate BVE7 based on 16S rRNA gene sequencing. The bootstrap values on the branching nodes were calculated on 1,000 replications.

BVE7 was a gram-positive, rod-shaped bacterium, measuring 0.4 to 0.8 μm in diameter and 1.5 to 3.4 μm in length, with round cell ends (Figure 2) and usually no fold and forms milky-white colonies on LB agar plates. With aging, the colony margins of BVE7 exhibited a folded morphology with a depressed surface. It was aerobic, and could produce nitrate reductase and hydrolyze starch and casein. M.R., amylum hydrolase and V-P tests were positive. BVE7 could utilize a variety of sugars, including sucrose, glucose, arabinose, mannitol, seminose lactose, xylose, phaseomannite, galactose, maltose, rhamnose, cellobiose and fructose, but not malonate. The bacterium did not produce hydrogen sulfide and catalase. The 16 s rRNA gene sequence of BVE7 showed 99.9% similarity to *B. velezensis* strain Bv-HR6-1 (accession no. MF192765.1) and was deposited in GenBank (accession no. OP905633.1). A phylogenetic tree based on the 16 s rRNA gene sequence comparison indicated that BVE7 belonged to the *B. velezensis* (Figure 3).

### 3.3. Effect of BVE7 on hyphal and conidia of *Fusarium oxysporum*

**Figure 4:**
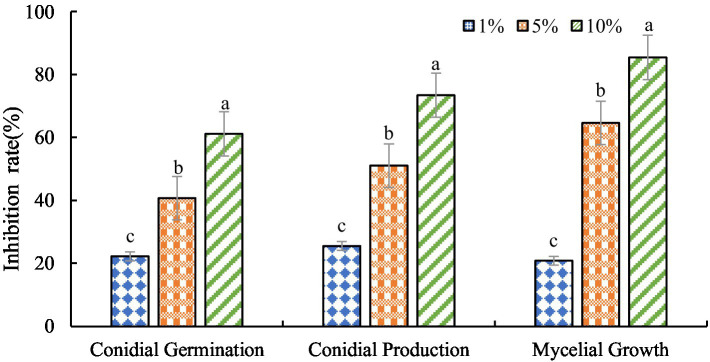
Effect of BVE7 filtrate on conidial germination, production and mycelial growth of *Fusarium oxysporum*. BVE7 filtrate was applied at concentrations of 1, 5, and 10%. Error bars indicate standard errors of the mean of two repeated experiments. Different letters above the bars indicate a significant difference within each group (i.e., conidial germination, conidial production, and mycelial growth) as determined by Duncan’s multiple range test (*p* < 0.05).

**Figure 5:**
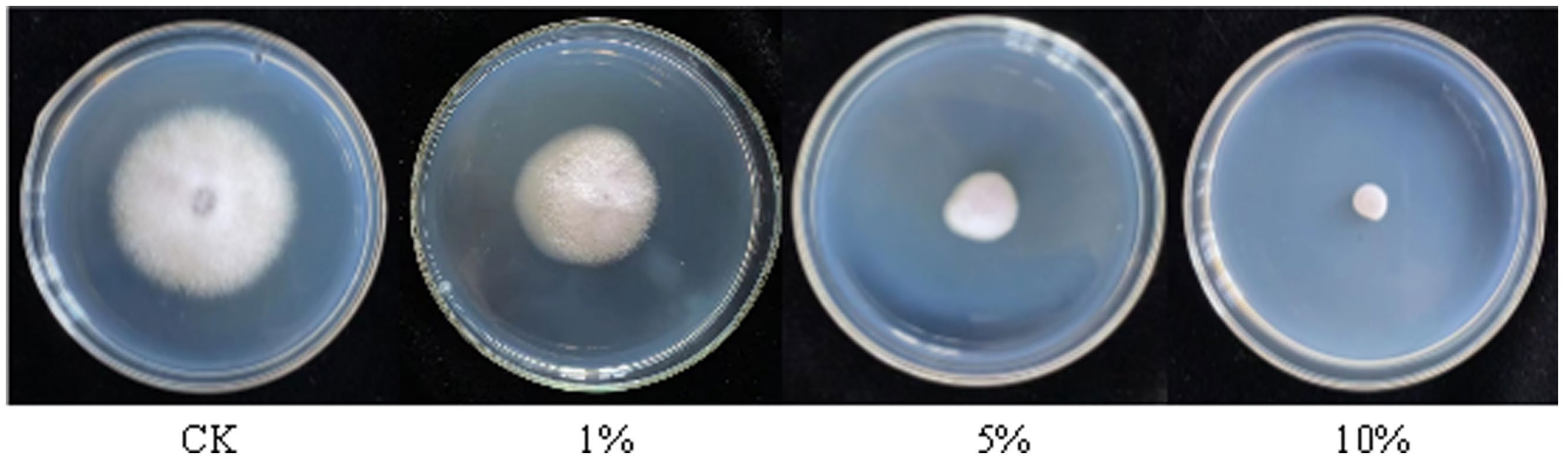
Effect of sterile fermented broth from BVE7 on *Fusarium oxysporum* mycelial growth on the fifth day after inoculation.

**Figure 6:**
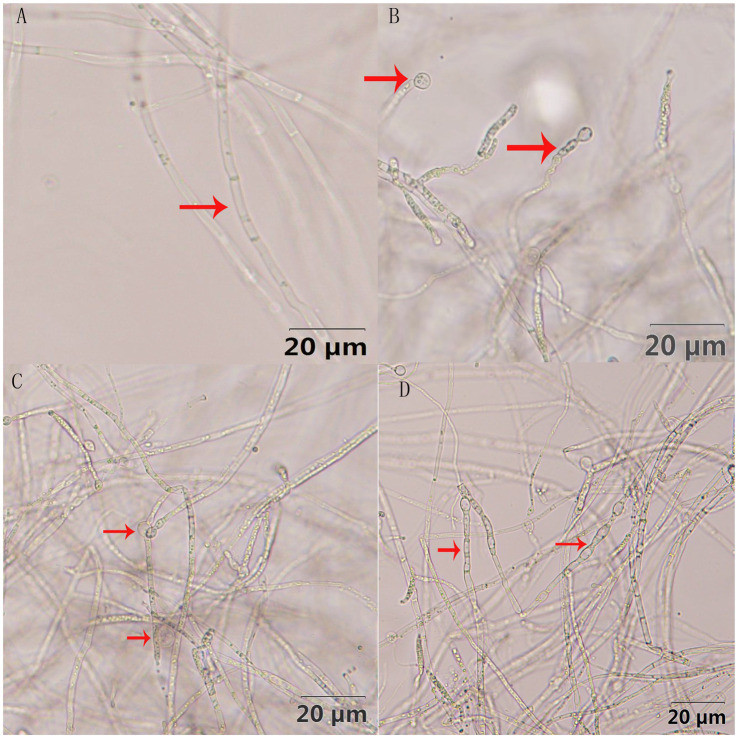
Effect of BVE7 filtrate on the mycelial morphology of *Fusarium oxysporum* (at a magnification of 400x). **(A)**, Non-treated control; **(B–D)** denote different concentrations (1, 5 and 10%, respectively) of treatment.

The antagonistic active components of BVE7, administered at varied concentrations, significantly inhibited the conidial production, germination, and hyphal growth of *F.* oxysporum (Figures 4, 5). At a concentration of 10%, BVE7 demonstrated a 61.1% inhibition rate on spore germination, 73.4% on spore production, and 85.42% on hyphal growth. Additionally, treatment with the BVE7 filtrate resulted in protoplasmic aggregation, swelling deformation, and folding of the *F. oxysporum* mycelium (Figure 6). As the concentration of BVE7 filtrate increased, these effects became more prominent.

### 3.4. Stability of the antifungal substances of BVE7

**Figure 7:**
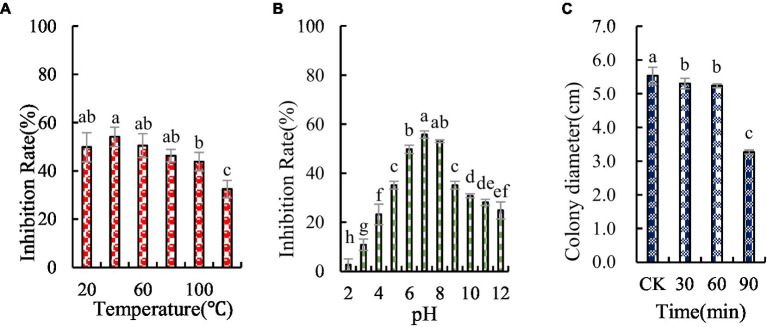
Stability of antifungal compounds in BVE7 under varied temperature regimes **(A)**, pH values **(B)**, and durations of ultraviolet exposure **(C)**. Error bars indicate standard errors of the means of two repeated experiments. CK was not exposed to UV. Different letters above the bars indicate significant difference within each treatment group (i.e., temperature, pH value, and ultraviolet treatment time) according to Duncan’s multiple range test (*p* < 0.05).

The antifungal substances in BVE7 were found to be unaffected by temperatures up to 60°C, but were significantly weakened at temperatures exceeding 80°C, though some antifungal activity persisted (Figure 7A). The antifungal substances were also observed to be pH-sensitive, with maximal activity at a pH of 7.0 (Figure 7B). Prolonged exposure to UV radiation was observed to enhance the antagonistic effect (Figure 7C).

### 3.5. Reduction SRR following application of BVE7

As shown in Table 3, the disease index of SRR when inoculated solely with *F. oxysporum* was around 70. When applied to a bacterial suspension of 1 mL per plant and 0.5 mL per plant, the control efficacy of SRR surpassed 65%. However, when treated with BVE7 at a bacterial suspension of 1.5 mL/plant, the control efficacy of SRR exceeded 75% when compared to the control group, which was equivalent to that of chemical fungicides.

**Table 3:** Evaluation of the efficacy of biological bacteria BVE7 suspension preventing soybean root rot caused by *Fusarium oxysporum* in pot experiments.

No.		Treatment	Disease index (%)^a^	Disease reduction (%)
1	i	Ck	0.0 ± 0.0e	–
	ii	*F. oxysporum* only	70.8 ± 2.9a	–
	iii	BVE7 suspension (0.5 mL/plant)	22.2 ± 1.2 b	68.3
	iv	BVE7 suspension (1 mL/plant)	20.1 ± 1.0c	71.2
	v	BVE7 suspension (1.5 mL/plant)	17.4 ± 1.3d	75.1
	vi	43% prochloraz(0.14 mg/mL)	18.2 ± 1.1d	74.0
2	i	Ck	0.0 ± 0.0e	–
	ii	*F. oxysporum* only	68.9 ± 1.8a	–
	iii	BVE7 suspension (0.5 mL/plant)	21.6 ± 0.9b	68.7
	iv	BVE7 suspension (1 mL/plant)	18.4 ± 1.1c	73.3
	v	BVE7 suspension (1.5 mL/plant)	16.2 ± 0.9d	76.5
	vi	43% prochloraz(0.14 mg/mL)	16.8 ± 1.2d	75.6

a Values in the column indicate Mean ± standard error (SE).

Values followed by different letters were significantly different according to Duncan’s multiple range tests (*p* < 0.05).

### 3.6. Effects of BVE7 treatment on the activities of defense enzymes in soybean root tissues

**Figure 8:**
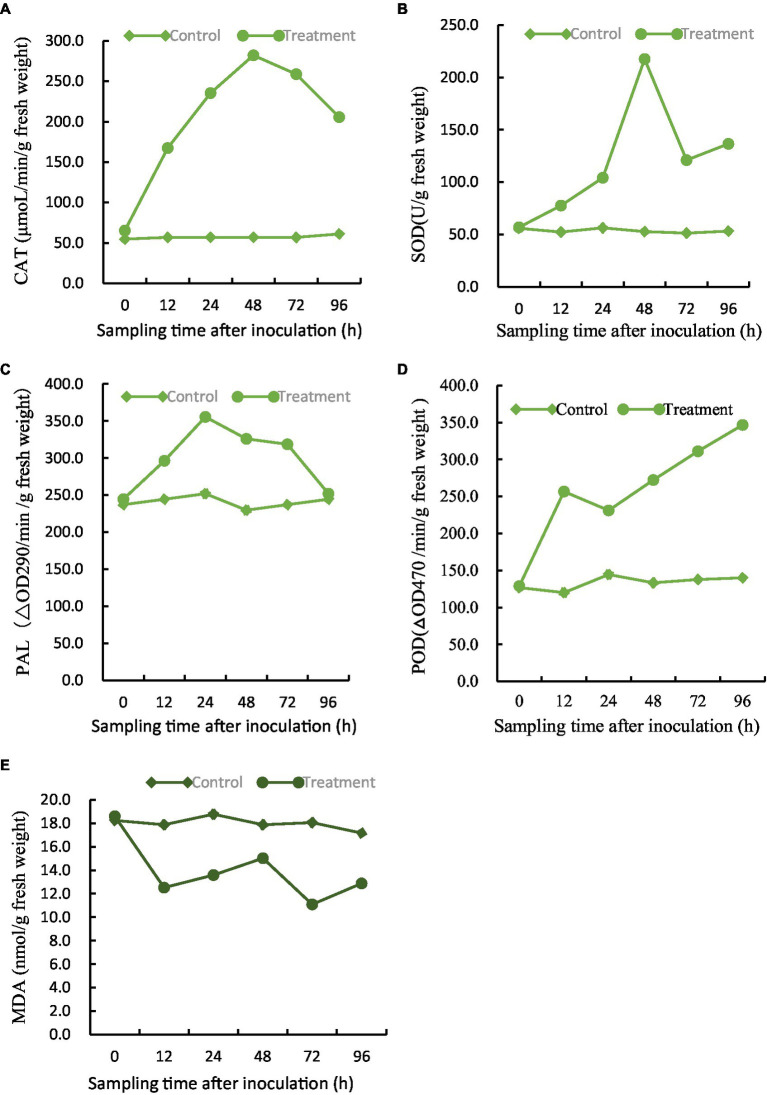
Effect of BVE7 on **(A)**, catalase (CAT), **(B)**, superoxide dismutase (SOD), **(C)**, phenylalanine ammonia-lyase (PAL), **(D),** peroxidase (POD), **(E)**, malondialdehyde (MDA). Two treatments with 3 replicates were included: **i**, 3 mL of sterile water as a control; **ii**, 1.5 mL suspension (1 × 10^8^ CFU/mL) of BVE7/each plant. Error bars indicate standard errors of the means of two repeated experiments. Different letters above the bars indicate significant differences (*p* < 0.05).

The efficacy of five major defense-associated enzymes in soybean roots treated with BVE7 was evaluated. The results showed that that CAT, SOD, and PAL activities exhibited an initial increase within 24 to 48 h, followed by a subsequent decline. Notably, treatment with BVE7 resulted in significantly higher activity levels than those observed in the control group (Figures 8A–C). Within 96 h, POD demonstrated a consistent upward trend, surpassing that of the control group by a significant margin (Figure 8D). MDA demonstrated slight fluctuations over a span of 96 h, yet remained significantly lower than the control group (Figure 8E).

In conclusion, soybean root treated by BVE7 activated the defense enzyme system to improve the disease-resistance.

## 4. Discussion

SRR, caused by *Fusarium oxysporum*, is the most important soil-borne disease in various soybean growing regions around the world, seriously affecting the yield and quality of soybeans. The Northeast Black Soil Region in China is one of the “Four Great Black Soil Regions of the World” and the “Three Great Cold Region Black Soil Regions.” The climate characteristics of this region are also one of the important reasons for the serious occurrence of SRR. At the same time, the cold environment results in significant differences in microbial species and biological control compared to other regions. Therefore, this study, based on isolation and application under cold conditions, has greater practical value for the prevention and control of SRR in the northern cold regions.

Biological control has attracted extensive attention due to its safety, environmental friendliness, and sustainability (Wei et al., 2023). Most of the *Bacillus* spp. reported as effective biocontrol agents have been isolated from the rhizosphere, and occasionally from the phyllosphere, and are classified as members of the *B. subtilis* complex (Cawoy et al., 2011), which form populations on plant tissues and can colonize roots of different monocot and dicot plant species (Fan et al., 2011). Therefore, this study obtained a soil-isolated strain *B. velezensis* BVE7, which a good antagonistic effect on the dominant strain *F. oxysporum* causing SRR and also had a good broad-spectrum against various pathogens causing SRR. *B. velezensis* has been widely used in the prevention and control of soil borne diseases in crops, including *B. velezensis* against *F. oxysporum* causing *Panax ginseng* root rot (Wei et al., 2023), *F. oxysproum* caused strawberry fusarium wilt (Hong et al., 2022), *F. oxysproum* caused root rot in *Polygonatum cyrtonema* (Chi et al., 2019), late blight of potato caused by *Phytophthora infestans* (Mahendra et al., 2022). Therefore, the application value of *B. velezensis* BVE7 in controlling soil-borne SRR is significant.

In this study, we discovered that the BVE7 sterile filtrate at a 10% concentration effectively inhibited the spore germination, production, and mycelial growth of *F. oxysporum*, with rates of 61.11, 73.44, and 85.42%, respectively, while also inducing hyphal malformations.

Cuellar-Gaviria et al. (2021) demonstrated that *B. tequilensis* EA-CB0015 had the capacity to colonize banana leaf surfaces and produce lipopeptides that could significantly reduce the severity of black sigatoka disease. Wu et al. (2014) determined that both the fermentation broth and cell suspension of *B. amyloliquefaciens* NJZJSB3 were capable of fully protecting detached leaves of canola (*Brassica napus* L.) from *Sclerotinia sclerotiorum* infection, while also exhibiting effective antifugal properties through their toluene, phenol, and benzothiazole volatiles. Wei et al. (2023) inferred that *B. velezensis* YW17 inhibited *F. oxysporum* by secreting antifungal lipopeptides, proteins, and volatile substances, thereby indirectly protecting ginseng from pathogenic fungal infections. Furthermore, *B. amyloliquefaciens* FZB42, reclassified as *B. velezensis*, produced surfactin, fengycin, and bacillomycin D in the lettuce rhizosphere, enhancing the lettuce’s defense response against fungal pathogens (Chowdhury et al., 2015). Tahir et al. (2017) found that the volatile organic compounds (VOCs) produced by *B. subtilis* SYST2 significantly inhibited the growth and spore germination of plant fungal pathogens. Myo et al. (2019) found that *B. velezensis* NKG-2 produced VOCs that negatively impacted the growth of several plant fungal pathogens, including *Fusarium* spp., *Botrytis cinerea*, and *Alternaria alternata*. Some VOCs produced by BCAs could also promote plant growth and induce plant systemic resistance (Gao et al., 2017; Wu et al., 2019). *B. amyloliquefaciens* FZB42 exhibited antagonistic interactions with *F. graminearum*, a plant-pathogenic fungus that threatened the production and quality of wheat and barley globally (Gu et al., 2017). Among these, bacillomycin-D and fengycin were found to effectively inhibit the growth of *F. oxysporum* (Koumoutsi et al., 2004) and induce morphological changes in the plasma membranes and cell walls of *F. graminearum* hyphae and conidia (Gu et al., 2017). Culture filtrates from two strains of *B. velezensis* CE 100 not only induced abnormal mycelial development with reduction in pigment, but also caused hyphal deformations with swelling and bulging of the fungal pathogen. In addition, an antifungal dipeptide [cyclo(prolyl-valyl)], isolated from the culture of CE 100, exhibited concentration-dependent inhibition of conidial germination in *F. oxysporum* f. sp. *Lycopersici* and prolonged incubation periods, which resulted in irregular hyphal morphologies with swollen septa and disorganized cell contents in treatments with the dipeptide (Hwang et al., 2022). These findings are consistent with our research outcomes and indicate the need for future isolation and identification of antifungal compounds. Additionally, we will conduct further analysis on the fungicidal agents found in BVE7. The results of this study will provide a theoretical foundation for the development of more efficient, safe, and reliable biological agents.

Bacteria inhabiting the rhizosphere, which can colonize plant roots and confer advantageous outcomes on plant growth, are known as plant growth-promoting rhizobacteria (PGPR) (Karthikeyan et al., 2010). The colonization of the rhizosphere by PGPR enhances their ability to promote plant growth and health. PGPR possess the potential to promote plant growth, enhance legume plant nodulation with *Rhizobium* spp., and inhibit the growth of plant pathogens. Rhizobial inoculants have been utilized for disease management in peanut caused by *A. flavus* or *A. niger* (Ahmad et al., 2008; Moretti et al., 2008). These PGPR have the ability to synthesize a diverse array of antibiotics which are commonly linked to their efficacy in inhibiting the growth of plant pathogens. Meanwhile, a number of PGPRs are capable of producing enzymes, including chitinases, cellulases, glucanases, proteases, and lipases, which can hydrolyze portions of the cell walls of various pathogenic fungi (Remans et al., 2008; Majeed et al., 2015). Within the rhizosphere microbial communities, the predominant PGPR strains require investigation to determine their mechanisms for promoting growth, which can subsequently be utilized to develop and enhance related agricultural products (Wang et al., 2022). *B. velezensis* L-1 caused abnormal growth of the *Botryosphaeria berengeriana* mycelium, inciting defense-related enzyme expression in pears. Its inhibitory percentage of pear ring rot was observed to be 76.55% after 11 days post inoculation (Sun et al., 2017). Gao et al. (2017) found that *B. velezensis* provided protection to tomato plants against fungal pathogens, such as *Alternaria solani* and *Botrytis cinerea*. The study showed that BVE7 significantly reduced the disease index of SRR, exhibiting a control efficacy of over 75% at a bacterial suspension of 1.5 mL/plant in the pot experiments. Moreover, BVE7 aided in improving the activities of CAT, PAL, POD, and SOD while concurrently reducing MDA activity. Multiple studies have demonstrated that elevated levels of ROS-scavenging enzymes, antioxidant enzymes, and defense enzymes are indicative of increased disease resistance (Warabieda et al., 2020). Augmented levels of POD and PALserve to protect plant cells against pathogenic infection (Zhang et al., 2016; Zhu et al., 2021). CAT is considered an important antioxidant enzyme that prevents cellular damage by the action of free radicals(Gebicka and Krych-Madej, 2019). SOD is a crucial cellular antioxidant enzyme that converts superoxide free radicals into oxygen and hydrogen peroxide (H_2_O_2_), thereby protecting cells from oxidative damage (Singh, 2022). Meanwhile, MDA, a widely-used indicator of oxidative stress, provides insights into the degree of lipid peroxidation in plant membranes. Therefore, the BVE7 is able to effectively enhance the level of defense enzymes in soybean roots, resisting the invasion of Fusarium oxysporum to protect the roots and mitigate the severity of root rot.

Therefore, we conducted a thorough assessment of BVE7’s efficacy in managing SRR, indicating its potential utility as a means of controlling SRR in soybean cultivation.

## 5. Conclusion

In this study, a soil-isolated strain BVE7 was identified as *B. velezensis*, which exhibited broad-spectrum activity against various pathogens responsible for SRR. The BVE7 sterile filtrate, at a concentration of 10%, demonstrated significant antifungal activity, effectively inhibiting the conidial germination, production, and mycelial growth of *F. oxysporum* by 61.11, 73.44, and 85.42%, respectively, leading to hyphal malformations. The antifungal compound produced by BVE7 showed adaptability to a normal environment. In a pot experiment, the BVE7 suspension effectively controlled SRR, with the highest control efficiency of 75.13%. Furthermore, BVE7 can effectively stimulate the activation of soybean root’s plant protection defense enzymes to reduce the damage caused by fungi and the severity of SRR. In the next step, we will conduct in-depth research on the antimicrobial substances in BVE7 and longer growth stage test instead of just the seedling stage, aiming to provide materials for the development of new biocontrol agents against fungi causing SRR and strengthen applied research.

## Data availability statement

The datasets presented in this study can be found in online repositories. The names of the repository/repositories and accession number(s) can be found in the article/supplementary material.

## Author contributions

LS: Writing – original draft, Resources, Validation. WW: Writing – original draft, Data curation, Formal analysis, Investigation. XZ: Formal analysis, Writing – original draft, Methodology, Software. ZG: Formal analysis, Writing – original draft, Supervision, Validation. SC: Writing – original draft, Data curation, Investigation, Methodology. SW: Writing – original draft, Formal analysis, Funding acquisition, Resources. YL: Writing – original draft, Writing – review & editing.
